# Correction: Analysis of canthin-6-one alkaloids derived from *Eurycoma* spp. by micellar liquid chromatography and conventional high-performance liquid chromatography: a comparative evaluation

**DOI:** 10.1039/d3ra90044d

**Published:** 2023-05-09

**Authors:** Attapon Sakdamas, Fonthip Makkliang, Waraporn Putalun, Thaweesak Juengwatanatrakul, Tripetch Kanchanapoom, Seiichi Sakamoto, Gorawit Yusakul

**Affiliations:** a School of Pharmacy, Walailak University Nakhon Si Thammarat Thailand gorawit.yu@wu.ac.th gorawit.yu@mail.wu.ac.th +66-75-67-2839; b School of Languages and General Education, Walailak University Nakhon Si Thammarat Thailand; c Faculty of Pharmaceutical Sciences, Khon Kaen University Khon Kaen Thailand; d Faculty of Pharmaceutical Sciences, Ubon Ratchathani University Ubon Ratchathani Thailand; e Graduate School of Pharmaceutical Sciences, Kyushu University Higashi-ku Fukuoka Japan; f Biomass and Oil Palm Center of Excellence, Walailak University Nakhon Si Thammarat Thailand

## Abstract

Correction for ‘Analysis of canthin-6-one alkaloids derived from *Eurycoma* spp. by micellar liquid chromatography and conventional high-performance liquid chromatography: a comparative evaluation’ by Attapon Sakdamas *et al.*, *RSC Adv.*, 2023, **13**, 6317–6326, https://doi.org/10.1039/D2RA07034K.

The authors regret that the name of one of the authors (Fonthip Makkliang) was shown incorrectly in the original article. The corrected author list is as shown above.

The Royal Society of Chemistry apologises for these errors and any consequent inconvenience to authors and readers.

## Supplementary Material

